# Chemokines Profiling of Patients with Preterm Birth

**DOI:** 10.1155/2014/185758

**Published:** 2014-04-28

**Authors:** Piotr Laudanski, Adam Lemancewicz, Pawel Kuc, Karol Charkiewicz, Barbara Ramotowska, Malgorzata Kretowska, Elwira Jasinska, Grzegorz Raba, Katarzyna Karwasik-Kajszczarek, Janusz Kraczkowski, Tadeusz Laudanski

**Affiliations:** ^1^Department of Perinatology and Obstetrics, Medical University of Bialystok, Bialystok, 15-276 Podlasie, Poland; ^2^Department of Perinatology and Obstetrics, Medical University of Bialystok, Marii Sklodowskiej Curie 24a, 15-273 Bialystok, Poland; ^3^Faculty of Computer Science, Bialystok University of Technology, Bialystok, 15-351 Podlasie, Poland; ^4^Institute of Obstetric and Emergency Medicine, University of Rzeszow, Żurawica, 37-710 Podkarpackie, Poland; ^5^Department of Obstetrics and Pathology of Pregnancy, Medical University of Lublin, Lublin, 20-081 Lubelskie, Poland

## Abstract

*Introduction.* Nowadays it is thought that the main cause of premature birth is subclinical infection. However, none of the currently used methods provide effective prevention to preterm labor. The aim of the study was to determine the concentration of selected chemokines in sera of patients with premature birth without clinical signs of infection (*n* = 62), threatened preterm labor (*n* = 47), and term births (*n* = 28). *Method.* To assess the concentration of chemokines in the blood serum, we used a multiplex method, which allows the simultaneous determination of 40 chemokines per sample. The sets consist of the following chemokines: 6Ckine/CCL21, Axl, BTC, CCL28, CTACK/CCL27, CXCL16, ENA-78/CXCL5, Eotaxin-3/CCL26, GCP-2/CXC, GRO (GRO**α**/CXCL1, GRO**β**/CXCL2 and GRO**γ**/CXCL3), HCC-1/CCL14, HCC-4/CCL16, IL-9, IL-17F, IL18-BPa, IL-28A, IL-29, IL-31, IP-10/CXCL10, I-TAC/CXCL11, LIF, LIGHT/TNFSF14, Lymphotactin/XCL1, MCP-2/CCL8, MCP-3/CCL7, MCP-4/CCL13, MDC/CCL22, MIF, MIP-3**α**/CCL20, MIP-3-**β**/CCL19, MPIF-1/CCL23, NAP-2/CXCL7, MSP**α**, OPN, PARC/CCL18, PF4, SDF-1/CXCL12, TARC/CCL17, TECK/CCL25, and TSLP. *Results.* We showed possible implication of 4 chemokines, that is, HCC-4, I-TAC, MIP-3**α**, and TARC in women with symptoms of preterm delivery. *Conclusion.* On the basis of our findings, it seems that the chemokines may play role in the pathogenesis of preterm labor. Defining their potential as biochemical markers of preterm birth requires further investigation on larger group of patients.

## 1. Introduction

The incidence of preterm birth in developed countries has not changed for 40 years and it ranges from 6 to 13%, despite significant advances in perinatal care [1]. Premature birth is one of the major causes of morbidity and mortality in developed countries. However, not all women admitted to the hospital with preterm labor symptoms give birth prematurely. The high cost of caring for these patients should be taken into account [2]. Currently, it is believed that the main cause of premature births is subclinical infection, which leads to premature contractile activity and/or rupture of fetal membranes through different potential mechanisms [3–6]. Microbiological studies indicate that 25–40% of preterm births may be the result of infection [7]. Perhaps this percentage is higher, because intrauterine infection is difficult to diagnose by conventional methods of identification based on microbiological culture [8].

Asymptomatic infection of the amniotic cavity may initiate a cascade of inflammatory factors that stimulate the production of prostaglandins, which consequently lead to abnormal uterine contractile activity and irreversible changes in the cervix as well as structures of membranes.

Evaluation of preterm labor markers, which induce clinically silent inflammation, may increase the effectiveness of detection and treatment of preterm delivery [9]. For several years, chemokines have been of special interest to researchers because of their likely participation in the initiation of parturition [10, 11]. Chemokines are a family of small molecular weight cytokines, which are involved in leukocytes stimulation and chemotactic gradient determining. The increase of the chemokines concentration could be associated not only with infection but also with the mechanism of labor [12]. In our previous pilot study we found significantly lower concentrations of one of the tested chemokine MIP-3*β*/CCL19 in the group of patients who gave birth prematurely, compared to the women demonstrating symptoms of preterm labor but delivered at term [13].

The aim of the present study was to determine the concentration of selected, hitherto practically unexplored, group of chemokines in sera of women with premature birth without clinical signs of infection and patients with threatened preterm delivery.

## 2. Material and Methods

The study group consisted of patients who delivered at three Polish tertiary centers: Department of Perinatology of the Medical University of Bialystok, Poland, (recruitment between 2007 and 2013), Department of Obstetrics and Gynecology of District Hospital of Przemysl, Poland, (recruitment between 2009 and 2012), and Department of Obstetrics and Pathology of Pregnancy of Medical University of Lublin, Poland, (recruitment between 2012 and 2013). About 1800, 1500, and 1600 births per year, respectively, took place at these departments. Great proportion of patients admitted to the above mentioned centers from district hospitals, where they were treated with steroids or antibiotics and subsequently gave birth within 72 hours, were excluded from the study (see exclusion criteria below).

The study protocol was approved by the Local Ethical Committee of Medical University of Bialystok, Poland, and an informed consent was obtained from each patient.

Biochemical studies were performed on serum samples from three groups of women: group I—women with preterm birth between the 23rd and the 36th weeks of gestation (62 patients), group II—women with subjective symptoms of threatening preterm delivery (so-called false preterm labor), between the 23rd and the 36th weeks, who delivered at term (47 patients), and group III—patients recruited at term who gave natural birth between the 39th and the 41st weeks of pregnancy (28 patients). The gestational age of all participating women was confirmed by ultrasound examination performed in the first trimester of pregnancy. The diagnosis of preterm labor (group I) was made according to previously established criteria [13, 14]. In all these patients, labor started with regular contractions and progressive cervical dilation (*n* = 29) or preterm premature rupture of membranes (pPROM)—(*n* = 33). The diagnosis of pPROM was defined as the presence of vaginal pooling with positive Amnisure test (Qiagen) prior to regular uterine activity.

Exclusion criteria from the study were multiple pregnancy, pregnancy induced hypertension, diabetes, kidney disease (creatinine above 2 mg/dL), and other complications during pregnancy, such as anemia, thrombocytopenia, systemic disease, thrombophlebitis, steroids and antibiotics within 72 hours of blood sampling, cervical incompetence and cervical cerclage, and finally clinical chorioamnionitis (at least one temperature elevation of >37.8°C, tachycardia, uterine tenderness greater than expected, white blood cell(WBC) count above 18,000, and unpleasant vaginal odor).

10 mL of peripheral blood was collected from each patient. The blood was then centrifuged; serum was separated and frozen at −80°C temperature.

To assess the concentration of chemokines in the blood serum we used a multiplex method, which allows the simultaneous determination of 40 chemokines per sample. Like a traditional sandwich-based ELISA, it uses a pair of specific chemokine antibodies for detection. A capture antibody is first bound to the glass surface. After incubation with the sample, the target chemokine is trapped on the solid surface. A second biotin-labeled detection antibody is then added, which can recognize a different isotope of the target chemokine. The chemokine-antibody-biotin complex is then visualized through the addition of the streptavidin-labeled Cy3 equivalent dye using a laser scanner (GenePix 4100A). Unlike the traditional ELISA, Quantibody products use array format. By arraying multiple chemokine specific capture antibodies onto a glass support, multiplex detection of chemokines in one experiment is made possible.

The sets (Quantibody Array Human Chemokine, RayBiotech Inc.) consist of the following chemokines: CC chemokine ligand 21 (6Ckine/CCL21), protein tyrosine kinase (Axl), betacellulin (BTC), chemokine (C-C Motif) ligand 28 (CCL28), cutaneous T-cell attracting chemokine (CTACK/CCL27), chemokine (C-X-C motif) ligand 16 (CXCL16), epithelial neutrophil-activating protein 78 (ENA-78/CXCL5), eotaxin-3/CCL26, granulocyte chemotactic protein 2 (GCP-2/CXC), growth-regulated protein *α*, *β*, *γ* (GRO*α*/CXCL1, GRO*β*/CXCL2, and GRO*γ*/CXCL3), hemofiltrate cc chemokine 1 (HCC-1/CCL14), hemofiltrate CC chemokine 4 (HCC-4/CCL16), interleukin 9 (IL-9), interleukin 17F (IL-17F), interleukin 18 binding protein (IL18-BPa), interleukin 28A (IL-28A), interleukin 29 (IL-29), interleukin 31 (IL-31), Interferon Inducible Protein 10 (IP-10/CXCL10), Interferon-Inducible T-cell alpha chemoattractant (I-TAC/CXCL11), leukemia inhibitory factor (LIF), ligand for herpesvirus entry mediator (LIGHT/TNFSF14), lymphotactin/XCL1, monocyte chemoattractant protein 2 (MCP-2/CCL8), monocyte chemoattractant protein 3 (MCP-3/CCL7), monocyte chemoattractant protein 4 (MCP-4/CCL13), macrophage-derived chemokine (MDC/CCL22), macrophage migration inhibitory Factor (MIF), macrophage inflammatory protein-3-alfa (MIP-3*α*/CCL20), macrophage inflammatory protein-3-beta (MIP-3*β*/CCL19), myeloid progenitor inhibitory factor 1 (MPIF-1/CCL23), neutrophil-activating peptide 2 (NAP-2/CXCL7), macrophage stimulating protein alpha (MSP*α*), Osteopontin (OPN), pulmonary and activation-regulated chemokine (PARC/CCL18), platelet factor 4 (PF4), stromal cell-derived factor-1 (SDF-1/CXCL12), thymus and activation regulated chemokine (TARC/CCL17), thymus-expressed chemokine (TECK/CCL25), and thymic stromal lymphoprotein (TSLP).

We also performed CRP (C reactive protein) and PCT (procalcitonin) determination. CRP was measured using immunoturbidimetric method with the Multigent CRP Vario assay (detectable range was 0.2–480 mg/L) detected on the ARCHITECT ci4100. PCT was assessed by the use of VIDAS B*·*R*·*A*·*H*·*M*·*S PCT test based on ELFA (enzyme linked fluorescent assay) technology (detectable range was 0.05–200 ng/mL).

Analysis of chemokines concentrations in each group was performed using GraphPad Prism package and STATISTICA 10. The analysis used nonparametric tests, due to rejection of the hypothesis of normal distribution of individual markers in groups (Shapiro-Wilk test). The Kruskal-Wallis test was used to compare the median concentration of chemokines between three distinguished groups, and differences at the significance level 0.05 were the basis for performing multiple comparisons with Dunn's test. We also used Kruskal-Wallis test to compare serum concentration of WBC, CRP, and PCT between three groups. The correlation between WBC, CRP, PCT, and serum chemokines concentration was assessed by Spearman rank correlation analysis.

Mann-Whitney-Wilcoxon's test was used to compare the median between the groups of patients who delivered before and after 7 days from the onset of signs and between the groups of patients with and without pPROM.

Receiver operating characteristic (ROC) curves were determined for statistically significant results between the groups of preterm labor and false preterm labor. The ROC curve describes the relationship between sensitivity (fraction of true positives) and the value of 1 − specificity (fraction of true negatives). The area under the curve (AUC) indicates the quality of a given characteristic as a classifier and the value of 0.5 indicates randomness of the test.

## 3. Results

Clinical characteristic of the patients is presented in Table 1. The median values of maternal serum chemokines concentrations in each study group and *P* values are presented in Table 2.

Patients with preterm labor had higher serum concentrations of 3 chemokines: I-TAC, MIP-3*α*, and TARC and lower serum concentration of HCC-4 (Table 2) when compared to patients with threatened preterm labor. We included these chemokines in later analyses and created ROC curves for them, which set the threshold values and allowed predicting the likelihood of preterm delivery with specific sensitivity and specificity (minimal sensitivity was set to 0.7).

**Figure 1:**
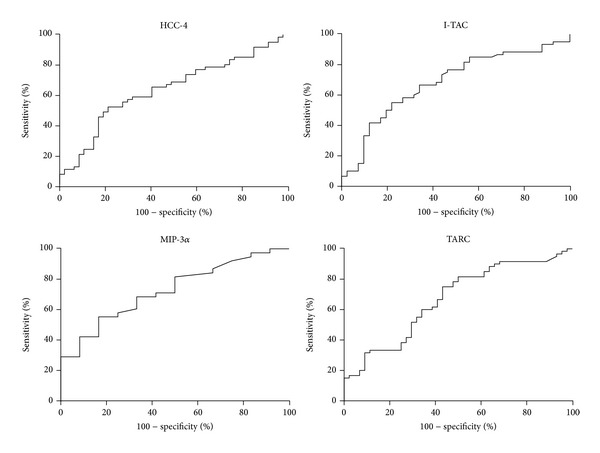
The ROC curves for concentration of chemokines HCC-4, I-TAC, MIP-3*α*, and TARC.

The area under the ROC curve for HCC-4 was 0.64, for I-TAC it was 0.68, for MIP-3*α* it was 0.73, and for TARC it was 0.67 (Figure 1). All field values are satisfactory and indicate the usefulness of these biochemical markers as tools to predict the risk of preterm delivery.

**Figure 2:**
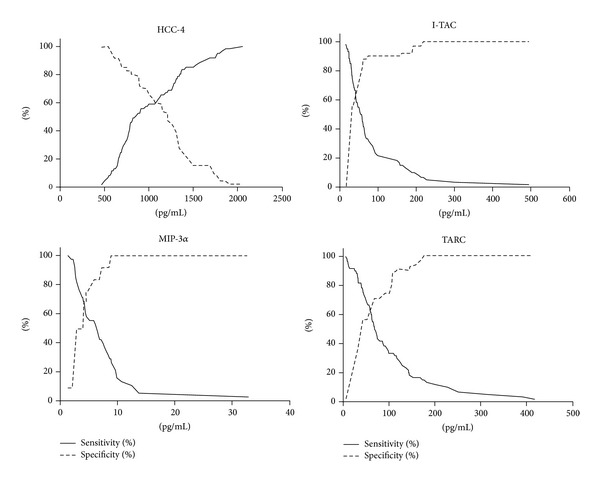
Sensitivity and specificity of markers HCC-4, I-TAC, MIP-3*α*, and TARC.

We demonstrated a significantly higher risk of preterm birth when the serum concentration of HCC-4 < 1285 pg/mL (sens. 0.74, sp. 0.45, *P* = 0.013), I-TAC > 33.35 pg/mL (sens. 0.73, sp. 0.56, *P* = 0.002), MIP-3*α* > 3.60 pg/mL (sens. 0.74, sp. 0.50, *P* = 0.019), and TARC > 44.10 pg/mL (sens. 0.75, sp. 0.57, *P* = 0.003) (Figure 2).

Diagnostic values of these chemokines are presented in Table 3.

When we compared the serum concentration of chemokines between patients with preterm labor before (*n* = 44) and after (*n* = 18) 7 days from the onset of signs and between patients with pPROM (*n* = 33) and without pPROM (*n* = 29), we did not observe statistically significant differences as to any of the studied chemokines.

We did not find any statistically significant differences when we compared serum concentration of WBC, CRP, and PCT between three groups using Kruskal-Wallis test. Statistically significant correlation, by Spearman rank correlation analysis, was identified only in the group of preterm labor between CRP and TECK: *r* = −0.31; *P* = 0.05 and between WBC and NAP-2: *r* = 0.46; *P* = 0.01.

## 4. Comment

In our present study, we examined the serum concentration of 40 chemokines in blood serum of pregnant patients and 4 of them were statistically significantly different between the groups of preterm labor and false preterm labor. The concentration of 3 chemokines, that is, I-TAC, MIP-3*α*, and TARC above the cut-off value and HCC-4 below the cut-off value, indicates the risk of preterm delivery.

In our previous work, we compared, among others, the serum concentration of such chemokines as I-TAC, MIP-3*α*, TARC, MIP-3*β*, MDC, and IP-10 between the groups of preterm birth (*n* = 17), false preterm labor (*n* = 13), and term deliveries (*n* = 8) [13]. We did not find statistically significant differences in chemokines I-TAC, MIP-3*α*, and TARC, which were found in the present study. However, this difference occurred in the chemokine MIP-3*β*. It could be due to the smaller number of patients who participated in the previous study. The concentrations of MDC and IP-10 did not show statistically significant differences in both studies.

There are potential explanations for the role of differentially expressed chemokines in the pathophysiology of preterm labor. I-TAC, as a factor of leukocytes migration, attracts active lymphocytes in the place of inflammation. It was found that I-TAC concentration in the amniotic fluid increases in the second trimester of pregnancy, in the period preceding the development of preterm delivery [10]. The increased concentration of I-TAC and MCP-4 was shown in children, who were born after preterm labor, membrane rupture, placental abruption, and cervical insufficiency [15]. These complications are likely to lead to fetal inflammatory response. In our study we confirmed that only I-TAC might play a role in predicting preterm delivery.

The concentrations of two studied chemokines, that is, MIP-3*α* and TARC, were higher in the groups of preterm labor and those who delivered at term as compared with false preterm labor. Previous studies also showed increased MIP-3*α* concentration during spontaneous labor at term and preterm pregnancies, what suggests its participation in the pathogenesis of childbirth. However, these two chemokines were also shown to be increased during infection [3, 16]. It gives the assumption that MIP-3*α* and TARC might be involved in the inflammatory process and be potential markers of silent inflammation during pregnancy.

The concentration of HCC-4 is significantly different between three studied groups—the concentration was the highest in the group of false preterm labor, lower in preterm births, and the lowest in term pregnancies. In previous studies, increased HCC-4 was related to preeclampsia, fetal growth restriction [17], and term births [18]. Higher concentration of HCC-4 was also demonstrated in proliferative endometrium than in atrophic endometrium [19]. This might indicate that chemokines do not only participate in the inflammatory process, but also affect other processes.

When we tested the concentration of total GRO, which consisted of three subunits GRO*α*/CXCL1, GRO*β*/CXCL2, and GRO*γ*/CXCL3, we found a significantly lower concentration in both preterm labor and false preterm labor as compared to natural term delivery. In one of the previous studies only GRO*α* subunit was tested in amniotic fluid and higher concentrations of this chemokine had been found during infection [20]. This relationship was not confirmed in a later study [21]. It is not known what might be the cause of decreased levels of certain chemokines in the group of preterm deliveries. It is possible that lower concentration may predispose to infection and chorioamnionitis, which seems to be a potential cause of premature birth [14]. It is necessary to perform further studies to investigate the possible role of these chemokines in premature childbirth.

It has been shown that chemokines could be potential markers of preterm delivery and might play a role in the pathology of premature delivery. Despite negative clinical and laboratory parameters of infection, we cannot rule out silent chorioamnionitis, which can affect the concentration of chemokines. We do not know whether the change of concentration of chemokines was associated with latent infection or the mechanism of labor. Further studies are required to determine the exact role of chemokines in preterm birth.

## Figures and Tables

**Table 1:** Clinical characteristic of the patients.

	Group I preterm labor (*n* = 62)	Group II false preterm labor (*n* = 47)	Group III births at term (*n* = 28)
Maternal age (mean ± SD)	28.17 ± 6.39	28.74 ± 5.54	27.73 ± 4.15
Number of pregnancies (mean ± SD)	2.032 ± 1.47	1.738 ± 1.01	1.679 ± 0.77
Gestational age at collecting of samples in weeks (mean ± SD)	29.97 ± 3.76	31.98 ± 3.34	39.25 ± 1.74
Gestational age at birth in weeks (mean ± SD)	31.45 ± 3.65	39 ± 1.57	39.68 ± 1.57
Body mass of newborn in grams (mean ± SD)	1866 ± 745	3143 ± 580.7	3556 ± 447.4
Present body mass in kg (mean ± SD)	66.69 ± 11.82	70.23 ± 13.63	77.14 ± 11.29
Body mass before pregnancy in kg (mean ± SD)	58.43 ± 9.98	60.58 ± 15.38	61.35 ± 9.59

**Table 2:** Concentration of chemokines in maternal sera.

	Group I— preterm labor *n* = 62	Group II— false preterm labor *n* = 47	Group III— births at term *n* = 28	*P* value*	Dunn's test***
	Chemokines concentration (pg/mL)			Group I—Group II—Group III
	Median (min–max)		
6Ckine	11539 (2713–77761)	12543 (3226–39407)	13199 (3328–74956)	0.47	
Axl	1306 (529.5–7509)	1389 (601.4–3074)	1278 (554.9–2203)	0.96	
BTC	8924 (5577–32195)	8246 (5383–18495)	8754 (6103–38241)	0.4	
CCL28	13504 (7193–41772)	13959 (7923–78508)	14449 (9270–54187)	0.43	
CTACK/CCL27	3831 (1028–93251)	4138 (1006–25364)	4205 (1932–27000)	0.88	
CXCL16	2849 (1789–7924)	2844 (1260–13175)	2975 (1976–13875)	0.62	
ENA-78/CXCL5	2894 (380.8–16116)	2728 (799.4–19843)	3999 (1187–12908)	0.06	
Eotaxin-3	4545 (2229–18529)	4269 (1507–23502)	4845 (3239–19933)	0.28	
GCP-2	709.7 (132.1–2507)	743.8 (190–1841)	649.8 (90.1–1334)	0.35	
GRO *α*. *β*. *γ*/CXCL1. CXCL2. CXCL3	522.6 (351.4–1187)	464.2 (350.7–891.9)	662.7 (485.1–1686)	<0.0001**	II-III I–III
HCC-1/CCL14	1246 (394.7–3547)	1302 (338.9–3414)	1197 (868.4–2146)	0.59	
HCC-4/CCL16	849.2 (452.5–1939)	1222 (550.6–2183)	710.7 (533.9–918.3)	<0.0001**	I-II II-III I–III
IL-9	87505 (41779–323057)	93656 (39866–148290)	94488 (65506–175965)	0.17	
IL-17F	1685 (81–25311)	1204 (99.7–20224)	1335 (138.3–17388)	0.40	
IL-18 BPa	9087 (2811–28743)	10040 (4294–25752)	11219 (6197–41799)	0.15	
IL-28A	279.8 (31.9–1778)	234.6 (43.2–481)	342.3 (42.4–525.6)	0.04**	II-III
IL-29	26560 (10546–105551)	24315 (11423–193283)	30091 (15078–131083)	0.02**	II-III
IL-31	573 (64.8–10512)	721.5 (64.8–31428)	474.3 (84.5–33119)	0.67	
IP-10/CXCL10	832.4 (366.8–1936)	985.3 (399.4–2013)	862.0 (524–1256)	0.08	
I-TAC/CXCL11	55.65 (14.7–620.5)	31.60 (18.8–215.4)	48.65 (14.1–255.2)	0.005**	I-II
LIF	2368 (378.4–4919)	2894 (814.3–4176)	2689 (1198–3678)	0.39	
LIGHT/TNFSF14	149.8 (42.8–768.7)	149.7 (48.7–315.8)	171.4 (82.1–259.4)	0.13	
Lymphotactin/XCL1	2814 (1457–9097)	2542 (1070–5303)	2918 (1180–4593)	0.50	
MCP-2/CCL8	7.9 (1.9–75.6)	6 (1.3–66.9)	11.55 (3.9–17.4)	0.08	
MCP-3/CCL7	54.35 (14.8–657.5)	43.7 (9.6–125)	50.7 (22–448.3)	0.36	
MCP-4/CCL13	120.5 (40.7–449.1)	96.3 (14.5–216.7)	118.2 (19–313.4)	0.08	
MDC/CCL22	3299 (778.8–9025)	3578 (152.3–9297)	3751 (1736–5840)	0.28	
MIF	365.5 (100.3–3214)	330.0 (105–5852)	460.9 (112.2–3347)	0.11	
MIP-3*α*/CCL20	6.33 (1.7–51.9)	3.35 (1–8.8)	6.8 (2.2–11.6)	0.03**	I-II II-III
MIP-3*β*/CCL19	889.5 (400.6–3476)	860.2 (327.6–11042)	963.2 (568–11621)	0.15	
MPIF1/CCL23	2670 (811.7–11855)	2540 (1003–4762)	2562 (1468–13547)	0.44	
MSP*α*	3780 (959.8–31556)	3801 (384.2–36637)	2935 (1014–15452)	0.13	
NAP-2/CXCL7	221.4 (103.8–614.7)	267.6 (125.5–614.7)	197.8 (141.6–324.8)	0.016**	II-III
OPN	255.2 (61.9–2243)	272.5 (49.7–703.8)	376.4 (86.8–954.5)	0.24	
PARC/CCL18	3217 (1073–7453)	2981 (1061–6378)	3122 (1621–4999)	0.67	
PF4	5137 (2564–24892)	5145 (1834–13097)	5251 (1684–11418)	0.81	
SDF-1/CXCL12	384.0 (45.7–1842)	329.5 (70.2–701.5)	333.5 (37.5–574.3)	0.52	
TARC/CCL17	68.40 (7.5–421.6)	39.25 (3.1–174.7)	98.25 (2.4–409.3)	0.002**	I-II II-III
TECK/CCL25	24077 (5484–52685)	22457 (5431–73659)	29067 (11315–41058)	0.0047**	II-III
TSLP	487.7 (174.1–4804)	395.2 (103.1–7183)	500.9 (108.6–2503)	0.33	

*P value for Kruskal-Wallis test.

**Statistically significant value of less than 0.05.

***Pairs of groups for which there are statistically significant differences (significance level 0.05).

**Table 3:** Diagnostic values of chemokines.

	Threshold value (pg/mL)	Sensitivity	95% CI for sensitivity	Specificity	95% CI for specificity	AUC	95% CI	Std. error	*P* value
HCC-4	<1285	73.77	60.93 to 84.20	44.68	30.17 to 59.88	0.64	0.53 to 0.74	0.05	0.013
I-TAC	>33.35	73.33	60.34 to 83.93	56.10	39.75 to 71.53	0.68	0.58 to 0.79	0.05	0.002
MIP-3*α*	>3.60	73.68	56.90 to 86.60	50.00	21.09 to 78.91	0.73	0.57 to 0.88	0.08	0.019
TARC	>44.10	75.00	62.14 to 85.28	56.82	41.03 to 71.65	0.67	0.56 to 0.77	0.05	0.003

## References

[1] Slattery MM, Morrison JJ (2002). Preterm delivery. *The Lancet*.

[2] Lucovnik M, Chambliss LR, Garfield RE (2013). Costs of unnecessary admissions and treatments for ‘threatened preterm labor’. *American Journal of Obstetrics & Gynecology*.

[3] Hamill N, Romero R, Gotsch F (2008). Exodus-1 (CCL20): evidence for the participation of this chemokine in spontaneous labor at term, preterm labor, and intrauterine infection. *Journal of Perinatal Medicine*.

[4] Menon R, Williams SM, Fortunato SJ (2007). Amniotic fluid interleukin-1*β* and interleukin-8 concentrations: racial disparity in preterm birth. *Reproductive Sciences*.

[5] Laudanski P, Raba G, Kuc P, Lemancewicz A, Kisielewski R, Laudanski T (2012). Assessment of the selected biochemical markers in predicting preterm labour. *Journal of Maternal-Fetal and Neonatal Medicine*.

[6] Kuć P, Laudański P, Kowalczuk O, Chyczewski L, Laudański T (2012). Expression of selected genes in preterm premature rupture of fetal membranes. *Acta Obstetricia et Gynecologica Scandinavica*.

[7] Goldenberg RL, Hauth JC, Andrews WW (2000). Intrauterine infection and preterm delivery. *The New England Journal of Medicine*.

[8] Relman DA, Loutit JS, Schmidt TM, Falkow S, Tompkins LS (1990). The agent of bacillary angiomatosis. An approach to the identification of uncultured pathogens. *The New England Journal of Medicine*.

[9] Laudanski P, Pierzynski P, Laudanski T (2007). Reductionist and system approaches to study the role of infection in preterm labor and delivery. *BMC Pregnancy & Childbirth*.

[10] Malamitsi-Puchner A, Vrachnis N, Samoli E (2006). Possible early prediction of preterm birth by determination of novel proinflammatory factors in midtrimester amniotic fluid. *Annals of the New York Academy of Sciences*.

[11] Wei S-Q, Fraser W, Luo Z-C (2010). Inflammatory cytokines and spontaneous preterm birth in asymptomatic women: a systematic review. *Obstetrics & Gynecology*.

[12] Haddad R, Tromp G, Kuivaniemi H (2006). Human spontaneous labor without histologic chorioamnionitis is characterized by an acute inflammation gene expression signature. *American Journal of Obstetrics & Gynecology*.

[13] Laudanski P, Lemancewicz A, Pierzynski P, Akerlund M, Laudanski T (2006). Decreased serum level of macrophage inflammatory chemokine-3*β*/CCL19 in preterm labor and delivery. *European Journal of Obstetrics & Gynecology and Reproductive Biology*.

[14] Simhan HN, Caritis SN, Krohn MA, de Tejada BM, Landers DV, Hillier SL (2003). Decreased cervical proinflammatory cytokines permit subsequent upper genital tract infection during pregnancy. *American Journal of Obstetrics & Gynecology*.

[15] Mcelrath TF, Fichorova RN, Allred EN (2011). Blood protein profiles of infants born before 28 weeks differ by pregnancy complication. *American Journal of Obstetrics & Gynecology*.

[16] Keelan JA, Khan S, Yosaatmadja F, Mitchell MD (2009). Prevention of inflammatory activation of human gestational membranes in an ex vivo model using a pharmacological NF-*κ*B inhibitor. *The Journal of Immunology*.

[17] Mäkikallio K, Kaukola T, Tuimala J, Kingsmore SF, Hallman M, Ojaniemi M (2012). Umbilical artery chemokine CCL16 is associated with preterm preeclampsia and fetal growth restriction. *Cytokine*.

[18] Kaukola T, Ojaniemi M, Tuimala J (2011). Cord blood chemokines differentiate between spontaneous and elective preterm births in singleton pregnancies. *Cytokine*.

[19] Jones RL, Hannan NJ, Kaitu’u TJ, Zhang J, Salamonsen LA (2004). Identification of chemokines important for leukocyte recruitment to the human endometrium at the times of embryo implantation and menstruation. *The Journal of Clinical Endocrinology & Metabolism*.

[20] Hsu C-D, Meaddough E, Aversa K, Copel JA (1998). The role of amniotic fluid L-selectin, GRO-*α*, and interleukin-8 in the pathogenesis of intraamniotic infection. *American Journal of Obstetrics & Gynecology*.

[21] Weissenbacher T, Laubender RP, Witkin SS (2013). Diagnostic biomarkers of pro-inflammatory immune-mediated preterm birth. *Archives of Gynecology and Obstetrics*.

